# Challenging the Role of Diet-Induced Anti-Neu5Gc Antibodies in Human Pathologies

**DOI:** 10.3389/fimmu.2020.00834

**Published:** 2020-06-09

**Authors:** Jean-Paul Soulillou, Emanuele Cozzi, Jean-Marie Bach

**Affiliations:** ^1^Centre de Recherche en Transplantation et Immunologie (CRTI), INSERM, Université de Nantes, and Institut de Transplantation Urologie Néphrologie (ITUN), CHU Nantes, Nantes, France; ^2^Department of Cardiac, Thoracic and Vascular Sciences, Transplant Immunology Unit, Padua University Hospital, Padua, Italy; ^3^IECM, Immuno-Endocrinology, USC1383, Oniris, INRAE, Nantes, France

**Keywords:** NeuGc, anti-Neu5Gc, autoantibodies, endothelial cells, epithelial cells, atheroma, cancers, humans

## Introduction

The thematic issue of *Frontiers in Immunology*, entitled “Human Antibodies Against the Dietary Non-Human Neu5Gc-Carrying Glycans in Normal and Pathological States,” benefits from an extensive fundamental review of previous studies on N-glycolylneuraminic acid (Neu5Gc) in humans [(1) for recent review and citations therein] and from studies on anti-Neu5Gc antibodies (A-GcAbs) in *Cmah*^−/−^ mice that, similar to humans, lack a functional cytidine monophosphate N-acetylneuraminic acid hydroxylase (CMAH), thereby allowing Neu5Gc synthesis (2). Further, “xenosialitis” assumes that the local interaction of A-GcAbs with traces of Neu5Gc residue at the surface of certain human cells may result in a chronic activation of Neu5Gc-displaying cells, which would eventually generate atheroma [for endothelial cells (ECs)] and malignancies (for epithelia) (1, 3). However, not many studies have been performed to date in humans, and these studies have not yet unambiguously demonstrated that A-GcAb-related xenosialitis could contribute to major diseases in humans.

This opinion article aims to assess the available evidence-based background of the concept of a deleterious effect of xenosialitis on human diseases, and to suggest alternative working hypotheses for future studies. As previously mentioned, xenosialitis, which is restricted to human pathologies in this opinion article, has been proposed as a “logical” basis for deleterious effects that would result from *in situ* interactions of A-GcAbs, which are present in human sera and diet-derived Neu5Gc deposits on certain human cells (1). However, this framework assumes the paradox that evolution would have allowed such a dangerous confrontation. Results from studies on *Cmah*^−/−^ mice have been extensively reviewed (1). However, using CMAH-deficient mice—which do not benefit from the coevolution adaptation that followed the lack of Neu5Gc in humans— poses an issue, as xenosialitis models require exogenous immunizations to *elicit* A-GcAbs (4, 5), due to the difficulty of “humanizing” the mice with a Neu5Gc-rich diet. In addition, cases of “positive data” arguing for xenosialitis in animal models have required experimental designs that use mice which are prone to spontaneously developing endothelial injuries (5) [see also comments in (6, 7)].

### Basic Facts to Consider in Applying the Concept of Xenosialitis to Humans

The main differences regarding xenosialitis between humans and CMAH-deficient mouse models in terms of prevailing conditions are as follows: (1) the actual levels of loading among human cells with diet-derived Neu5Gc, and (2) the differences between A-GcAbs that result either from immunization by diet or from *active* immunization with animal-derived products and adjuvants.

#### Levels of Neu5Gc-Loading Among Human Cells With Diet-Derived Neu5Gc

The presence of Neu5Gc traces on ECs or epithelial cells from various organs in humans has been established using ten autopsy samples (8). Since unambiguously observing such deposits using anti-Neu5Gc chicken polyclonal Ab staining on frozen or fixed-histological tissue samples was difficult, we used flow cytometry to assess the binding of anti-Neu5Gc chicken Abs on living ECs from large arteries of brain-dead donors (9). Although we confirmed a faint signal on gated ECs in four samples, three other preparations were found to be negative (9). A roughly similar proportion was found in eight additional living EC preparations that were tested after sorting, of which, two were positive, two were negative, and four had extremely faint or negative staining (*unpublished data*). This is distinguished from the high-Neu5Gc loading among certain malignant cells (10), which may enable therapy using exogenously produced A-GcAbs and to monitor A-GcAb levels as a disease marker (11, 12). The low metabolic incorporation of Neu5Gc in humans with a Neu5Gc-rich diet can be explained by intestinal colonization of sialidases producing bacteria (13, 14).

Few studies have investigated Neu5Gc loading among human cells (8, 9); however, further studies are warranted to establish whether indeed a substantial fraction of humans actually lack the fundamental component of the theoretical basis of the xenosialitis model. Although Neu5Gc exists at trace levels on ECs of positive individuals, we must also consider the myriad of surface glycoproteins or lipoproteins that display Neu5Gc and the high diversity of Neu5Gc terminal residues, which result in a huge dispersion of cell-surface epitopes that are potentially recognized by A-GcAbs (15). It is thus possible that, following interactions with A-GcAbs, the coexistence of trace levels of antigens and the high epitope dispersion results in physiological cellular signaling that is below the activation threshold. In a recent analysis of affinity-purified natural A-GcAbs detected by ELISA and Glycan arrays, the authors suggested that “specific A-GcAbs” may only represent a small minority of the pool detected in the assays (16). These data confirm our working hypothesis. In addition, whatever the molecular definition of the glycans recognized by the natural, diet induced, anti-Neu5Gc measured by ELISA or Arrays, the question of their biological effects in humans remains.

Indeed, only a few *in vitro* studies explored the effects of A-GcAbs on human cells. The first (17) suggests there is an activation of umbilical ECs that develop a white blood cell binding phenotype after incubation with A-GcAbs-containing whole serum. However, these first experiments used several extra-physiological conditions; for instance, the Neu5Gc loading among ECs far exceeding the levels naturally observed in human ECs and the high anti-Neu5Gc titer of the serum tested. A second study (9) used affinity-purified A-GcAbs from either normal sera (diet-induced Abs) or sera of those highly immunized by rabbit polyclonal IgGs (elicited Abs) (18). In addition, large artery ECs that undergo physiological loading levels of Neu5Gc were used (9).

Although this last study (9) was restricted to the complete transcriptomic patterns and apoptosis of stimulated ECs, it is interesting that the activation patterns triggered either by purified diet-derived human A-GcAbs or by rabbit IgG-elicited A-GcAbs in these more physiological conditions did not present a classical “inflammation-like” activation of ECs. In contrast, the observed patterns are consistent with the concept that A-GcAbs may contribute to the homeostasis of ECs (9). Moreover, purified A-GcAbs were shown to downregulate classical inflammation patterns that are induced by the presence of normal sera, added as a complement source (with components also necessary to cell homeostasis) (9). Further, purified A-GcAbs inhibited important master genes involved in EC activation (9). In conclusion, the theoretical basis of xenosialitis in humans, which involves A-GcAbs, requires an improved assessment of the actual levels of Neu5Gc loading among human cells *in vivo* and of the percentage of normal individuals who exhibit detectable Neu5Gc on ECs or epithelia. In addition, the effects of purified A-GcAbs on ECs or epithelial cells should be tested *in vitro* under experimental conditions that more closely mimic “physiological” Neu5Gc loading.

#### Differences Between Anti-Neu5Gc Abs That Result From Immunization by Diet and Those Elicited by Active Immunization With Animal-Derived Products

Humans develop A-GcAbs within the first few months of life after being introduced to a Neu5Gc-containing diet (19). The impact of food antigens on immunity is poorly understood; further, the apoptosis of diet-activated T cells is a hallmark of the healthy intestine (20). Whether diet/microbiota levels significantly affect A-GcAb levels in healthy adults has not yet been determined (21). In contrast, after implantation of animal biodevices (22, 23) or infusion of animal-derived molecules, such as rabbit IgGs, blood-elicited A-GcAb levels drastically increase for several months (18) and largely exceed the average normal levels in non-immunosuppressed individuals. As expected, these “exogenously” elicited Abs display a high affinity and altered repertoire (24). In contrast to diet-derived natural immunization, the elicited responses result in a vigorous, memory-type induction of A-GcAbs in young adults (18) with a significant number of individuals exhibiting extremely high titres (from 20 μg/ml up to 1 g/l). The extent to which proportion-elicited A-GcAbs stemmed from B cells that were primed by diet-derived Neu5Gcs is currently unknown. Importantly, exposure to such high titres of A-GcAbs affects drug half-life and is associated with the serum sickness disease (SSD), likely due to the A-GcAbs (25). However, SSD is linked to immune complexes that circulate (26), rather than *in situ* xenosialitis. Whether the increase in late renal failure in those who develop SSD (25) results from early graft injury due to immune complexes, or xenosialitis that results in long-term exposure to elicited A-GcAbs, remains unknown. The late loss of transplant function that is associated with the highest elicited A-GcAb titres in patients who received rabbit IgGs [in Supplementary Data of (25)] is yet anecdotal, generated by a small group of patients in the absence of graft histological samples. There are thus two different contexts that must be considered. Deleterious-elicited A-GcAbs (as tested experimentally in CMAH-deficient mice) do not imply that diet-induced “natural” A-GcAbs are necessarily detrimental. Since extremely high titres of elicited A-GcAbs in non-immunosuppressed patients were not associated with even a clinically detectable acute vascular insult (27), coevolution adaptation to diet-induced A-GcAbs may also operate to control elicited A-GcAb effects. Similarly, diet-induced A-GcAbs within the first year of life are not associated with vascular pathology. Thus, along with the “threshold effect” hypothesis, the presence of protective mechanisms, which are likely shaped by evolution to escape the deleterious effects of A-GcAbs, is another working hypothesis to consider.

### A-GcAb Levels in Human Diseases—Particularly in Cases in Which Animal Models Suggested a Possible Role of Xenosialitis (Table 1)

Elevated A-GcAb levels have been determined to be inversely correlated with non-atopic asthma in farmers' children (32). Likewise, elevated A-GcAb titres have also been observed in patients with normal coronaries in Kawasaki disease (29). Both observations do not suggest a link between A-GcAb titres and inflammation.

**Table 1:** Clinical correlations between died-induced A-GcAbs and pathologies.

**Pathologies**	**Aim/rational**	**Clinical findings**	**Significance and limits**	**References**
Malignancy	Investigate whether anti-Neu5Gc antibodies increase the risk of colorectal cancer (CRC).	No correlation was found between antibodies against Neu5Gc alone or against individual Neu5Gc-bearing epitopes and CRC. However, a sialoglycan microarray study demonstrated a positive association of CRC risk and total anti-Neu5Gc-glycan antibody responses.	Whilst a correlation of CRC with the total anti-Neu5Gc antibody response has been demonstrated, a link with causation is still lacking.High Neu5Gc loading of malignant cells may also boost anti-NeuGc levels.	(4)
	Investigate whether anti-Neu5Gc elicited by rabbit IgGs are associated with a higher incidence of colon carcinoma.	There was no evidence that exposure to high levels of elicited anti-Neu5Gc antibodies is associated with a higher incidence of colon carcinoma.	This study relies on indirect evidence: ATG-treated renal allograft recipients have high-level anti-Neu5Gc antibodies.	(28)
Vascular diseases	Investigate if the levels of A-GcAbs correlate with chronic vascular lesions.	No correlation of A-GcAbs with coronary artery disease (CAD).	Case-control study using three tests for A-GcAbs (835 CAD vs. 1869 controls).	(21)
	Investigate if the levels of A-GcAbs correlate with acute vascular lesions.	No acute vascular pathology reported in young type 1 diabetic patients with extremely high titers of elicited A-GcAbs.	Deals with *elicited* A-GcAbs.	(18, 27)
		Inverse correlation of A-GcAbs and arterial lesions in Kawasaki disease.	Case-control study.No reported vascular pathologies.	(29)
		Increase A-GcAbs in acute EBV primo infection (IMN).	No reported vascular lesion in IMN.	(30)
Infertility	A-GcAbs could block the capacitation and migration of Neu5Gc-loaded spermatozoid, and egg fecundation and implantation in the female uterine tract exhibiting Neu5Gc.	No correlation between the presence of Neu5Gc or A-GcAbs in uterine tract and semen quality or uterine pathology.	Only a minority of men, even from infertile couples, incorporated Neu5Gc in sperm.Interesting hypothesis but limited number of cases studied yet.	(31)
Asthma	Investigate whether exposure to Neu5Gc is involved in the protection against allergy, asthma, and inflammatory bowel disease observed in children exposed to farm environment.	Farmers' children had elevated levels of anti-Neu5Gc antibodies that were inversely correlated with wheezing and asthma in non-atopic subjects.	Significant limit: the authors speculate that Neu5Gc behaves *in vitro* as an anti-inflammatory molecule in humans. However, free circulating Neu5C is controversial *in vivo*.	(32)
Multiple sclerosis	Possible effect on Brain Blood Barrier permeability	A-GcAb reactivity towards some Neu5Gc-bearing synthetic glycans in MS patients.	No increase of A-GcAbs in the blood of MS patients in another study.	(30, 33)

Xenosialitis has been proposed as a contributor to colon cancer, due to also being associated with high red meat intake (21). However, no association between A-GcAb levels, red meat intake, or coronary artery disease (CAD) risk, has been evidenced in adults, as assessed by several types of ELISA (21). In comparison, colorectal cancer (CRC) was found to be significantly associated with total A-GcAb responses using a Sialoglycan Microarray that measures the Ab repertoire against Neu5Gc-glycans (21). Nevertheless, the increased A-GcAb titres observed may merely represent an immunogenic marker of the strong Neu5Gc loading of malignant cells (10, 12, 34). Another study compared the incidence of colon cancer between kidney recipients (including 212,465 patients and 522 with colon cancer) who either received or did not receive rabbit anti-T cell IgGs that are able to induce long-term elevated A-GcAbs (28). While relying indirectly on inducing elicited A-GcAbs by rabbit IgGs in immunosuppressed patients (25), a long-term survey showed no difference in colon cancer incidence (28).

In further studies, the deleterious effects of A-GcAbs from females (and potentially males) have been reported on spermatozoids and egg implantation in the female uterine tract that contain Neu5Gc from diet, which thus may imply that xenosialitis is involved in certain cases of infertility (31). However, neither the presence of Neu5Gc nor that of A-GcAbs have been correlated with differences in semen quality or the presence of uterine pathology (31).

There are also several instances in the clinical arena in which a low antigen density on ECs does not result in identifiable deleterious effects, such as when blood group A2 organs are transplanted in ABO-incompatible recipients (35). Moreover, normal individual sera display a high diversity of Abs that cross-react with self-determinants (36, 37), which may provide anti-apoptotic signals and shape the immune repertoire by enabling more efficient cognitive responses. Significantly increased levels of A-GcAbs have also been reported in EBV acute infectious mononucleosis (IMN), which is likely related to the concomitant high percentage of EBV-infected B cells (30). As IMN is associated with high incidence of multiple sclerosis (MS) (38), it has been hypothesized that A-GcAb levels could enhance the migration of anti-EBV T cells through the blood-brain barrier (39). However, as previously mentioned, transcriptomic studies *in vitro* do not reveal patterns that are classically associated with EC inflammation (9). Using a semi-quantitative synthetic glycan array, a recent study reported a specific pattern of IgG reactivity for some Neu5Gc epitopes in MS patients compared to other neurologic diseases (33).

Thus, whether the concomitant traces of Neu5Gc on ECs and of diet-induced circulating A-GcAbs theoretically trigger inflammation at the site of the antigens, either by direct or complement-mediated effects, or by bridging CD16 positive blood mononucleated cells onto ECs, remains to be explored. Due to the absence of convincing and statistically-powered clinical evidence of xenosialitis, we recommend critically revisiting associated concepts and exploring the possibility that diet-derived A-GcAbs may contribute to EC homeostasis.

## Conclusion

As Galileo said, experiments are “questions asked to nature,” and so scientists routinely encounter subjectivity in their designs. We are aware that this limitation also exists when elaborating on the putative role of diet-induced “natural” A-GcAbs in the clinical arena, especially following Descartes' “*de omnibus dubitandum*” seminal warning. We suggest that a revisiting of the role of A-GcAbs in human biology with new tools and innovative working hypotheses will benefit scientific understanding and clinical application.

## Author Contributions

All authors thoroughly discussed all assertions of the correspondence and wrote this opinion paper.

## Conflict of Interest

J-PS and J-MB are cofounders of the Xenothera start-up. The remaining author declares that the research was conducted in the absence of any commercial or financial relationships that could be construed as a potential conflict of interest.

## Abbreviations

A-GcAbs: Anti-Neu5Gc antibodies
CAD: Coronary artery disease
CMAH: Cytidine monophosphate N-acetylneuraminic acid hydroxylase
CRC: Colorectal cancer
EBV: Epstein-Barr virus
IMN: Infectious mononucleosis
ECs: Endothelial cells
Neu5Gc: N-glycolylneuraminic acid
MS: Multiple sclerosis
SSD: Serum sickness disease.
